# *Babesia divergens*: A Drive to Survive

**DOI:** 10.3390/pathogens8030095

**Published:** 2019-07-02

**Authors:** Cheryl A Lobo, Jeny R Cursino-Santos, Manpreet Singh, Marilis Rodriguez

**Affiliations:** Department of Blood Borne Parasites, Lindsley F. Kimball Research Institute, New York Blood Center, New York, NY 100065, USA

**Keywords:** Babesia, invasion, malaria, persistence, population structure

## Abstract

*Babesia divergens* is an obligate intracellular protozoan parasite that causes zoonotic disease. Central to its pathogenesis is the ability of the parasite to invade host red blood cells of diverse species, and, once in the host blood stream, to manipulate the composition of its population to allow it to endure unfavorable conditions. Here we will review key in vitro studies relating to the survival strategies that *B. divergens* adopts during its intraerythrocytic development to persist and how proliferation is restored in the parasite population once optimum conditions return.

*Babesia* parasites present a complex life cycle spanning two hosts—a tick vector and a mammalian host. Despite this, the parasite has successfully established itself as the second most common blood-borne parasites of mammals after trypanosomes. The genus *Babesia* comprises more than 100 species of protozoan pathogens that infect erythrocytes of many vertebrate hosts [1]. They are transmitted by their tick vectors during the taking of a blood meal from the host [1,2]. Babesiosis has long been recognized as an economically important disease of cattle, but only in the last 30 years some species of *Babesia* have been recognized as important pathogens in man—*B. divergens* being one such agent. *B. divergens*, a natural pathogen of cattle, is the main pathogen of human babesiosis in Europe [3,4,5], with the majority of cases being reported in the British Isles and France [6]. This review article will focus on the key strategies adopted by *B. divergens* that allow it to successfully establish itself in diverse hosts, despite changing and hostile environments including the parasite’s multiple modes of transmission, its spread as a zoonotic agent, unique facets of its asexual life cycle within the RBC that allow it to alter parameters of the cycle, and, finally, parasite persistence in the face of hostile environments, including nutrient deprivation and toxic chemicals.

## 1. Modes of Transmission

**Figure 1:**
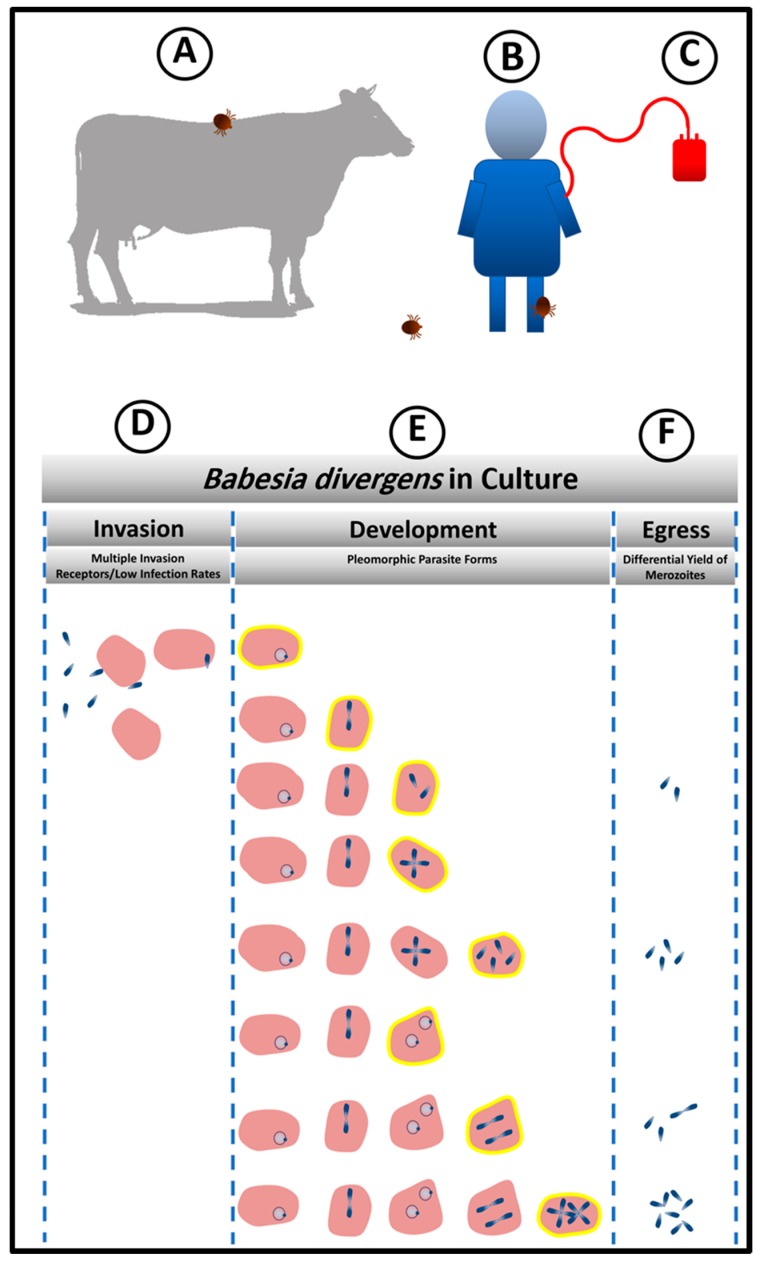
Persistence Model based on in vitro system. (**A**,**B**) Animals and humans get infected by the bites of infected ticks. (**C**) Babesiosis is also transmitted by blood transfusion. The storage of blood bags at low temperature reduces the size of Bd population. However, once under optimal condition of in vitro culture, Bd is capable of rebuilding its population from few parasites. (**D**,**F**) Biological processes that the parasite can control to promote persistence. (**D**) Control of invasion (**E**) From the top to the bottom, a complex population structure is built to gain heterogeneity which guarantee the provision of both the number of parasites and parasite stages, for prompt parasite response to environmental changes. Infected RBCs highlighted in yellow represent the diversity of stages and different parasite loads that can stay as the previous stage or keep proliferating within the same host cell to increase the reservoir of infecting individuals. (**F**) Parasite persistence is controlled by choice of host cells to be lysed and the number of parasites released during egress.

Babesiosis shows a worldwide distribution and affects a wide variety of many mammalian species. The natural and most important mode of parasite transmission is the bite of an infected ixodid tick (Figure 1). The only confirmed vectors of *Babesia* parasites are members of the Ixodidae family. *B. divergens* is transmitted by the *I. ricinus* tick, whose life-cycle is 3 years, as the larva, nymph, and adults each mature in a consecutive year. Most tick-borne infections are reported between April and October, which coincides not only with the warmer weather when ticks are more active, but also when individuals spend more time within tick-infested areas. Because Ixodidae ticks feed only once per instar [7], Babesia has developed the ability to persist through successive tick developmental stages, referred to as transstadial or transovarial transmission [8]. This allows for the spread of the parasite from a single maternal tick to thousands of offspring [8]. Transovarial transmission is considered to be an unusual mode of propagation within the phylum Apicomplexa. Within the order Piroplasmida, it is exclusively displayed by the evolutionary lineage of Babesia sensu stricto. The Babesia capacity for long-term occupation of ticks via transovarial transmission translates into parasite reservoirs even during the absence of the vertebrate host [9]. The proficiency of transmission, together with the worldwide distribution of tick vectors, makes Babesia the most common parasite of free-living and/or domestic animals [3]. As babesiosis became an important human medical condition, its persistence via other modes of transmission became apparent. Babesia can be secondarily transmitted via a blood transfusion with infected blood (Figure 1) [10,11,12], or even congenitally during pregnancy [13,14,15,16]. Worldwide, little attention has been given to transfusion-associated cases, but they likely occur in areas where babesiosis is endemic [17].

## 2. *Babesia divergens* as a Zoonotic Agent

The emergence of zoonotic diseases has been linked to major ecological changes facilitated by globalization and increased international travel and trade, climate change, habitat modifications, population growth, urbanization, agricultural intensification, and pathogen and vector evolution. A zoonosis is defined as any disease or infection that is naturally transmissible from vertebrate animals to humans. This transmission is facilitated by the Ixodes tick which as described earlier has a global distribution. Humans are accidental hosts of Babesia [6], but many aspects—for example, increasing travel activity, blood transfusions—contribute to human babesiosis as a serious public health concern. Human cases of babesiosis are difficult to quantify because many cases are not detected and diagnosed correctly, and others have not been reported or published. However, it is clear that in the last 75 years, the number of babesia cases have been steadily increasing, now being seen in new parts of the world and in greater numbers. The typical host reservoirs for *B. divergens* is cattle. Human disease due to babesia was first confirmed in Europe with the description of a fatal *Babesia divergens* infection in 1956 in the former Yugoslavia [18] and, ever since, babesiosis has been viewed as a potentially life-threatening zoonotic disease in humans [19,20,21]. Human babesiosis can persist in endemic areas by the occurrence of such multiple environmental life cycles involving the definitive (tick, where sexual reproduction occurs) and the intermediate (humans) hosts.

## 3. Features of the *Babesia divergens* Lifecycle that Dictate Parasitism Success

*B. divergens* has developed unique strategies in order to complete its life cycle along with efficient transmission and facilitate persistence of the parasite in the host. The critical reproductive aims of *B. divergens* are: (i) to continue its parasitic existence by propagation, and (ii) to guarantee host-to-host transmission through specialized infective stages [22]. Both objectives are mediated by the combination of asexual reproduction cycles and sexual reproduction cycles, which alternate between the vertebrate host and the tick vector. This review will focus primarily on the asexual erythrocytic stages of *B. divergens*. When *B. divergens* sporozoites are first injected into the human host, they target the host RBCs (Red blood cells) immediately, unlike *Plasmodium* spp. which are required to undergo an exo-erythrocytic phase in hepatic cells. Furthermore, infected RBCs remain circulating in peripheral blood stream, including regularly passing through the hosts’ spleen, and do not sequester to the fine capillaries of the bone marrow or organs. It is the parasite’s ability to first recognize and then invade host RBCs that is central to human babesiosis, and the parasites invade RBCs using multiple complex interactions between parasite proteins and the host cell surface, which are not fully elucidated yet [23,24,25,26,27,28,29]. Once inside the RBC, the parasite begins a cycle of maturation and growth exhibited by intense intra-cellular proliferation. The early stages of the cycle are morphologically indistinguishable from *Plasmodium* spp., with both appearing as ring-like parasites. Parasite egress is dictated by environmental conditions in terms of both the timing of this critical phase of the life cycle as well as the number of merozoites released from an infected RBC [30].

## 4. Efficient Invasion Is the First Step of Establishing Successful Parasitism

Once sporozoites from tick salivary glands enter the bloodstream of the human host, they invade erythrocytes (Figure 1B). It has not been experimentally ascertained as to how many sporozoites are inoculated by the tick bite. However, based on in vitro observations, not every parasite that is present in the inoculum invades the host RBCs. In fact, this is the first strategy of persistence that the parasite exploits in the host, by controlling the number of invaded RBCs as a way to protect the host and ensure his survival from the hemolysis that would result from a massive invasion (Figure 1D). Even with inoculum containing high numbers of free viable merozoites that have been mechanically released from RBC [30], the parasite initiates populations at low infection rates. In a 24 h period, while this number of infected cells does not increase much, some of the infected RBCs hosting single parasites transform into powerhouses of intense parasitic proliferation while remaining protected within the same host cells. The parasite thus builds a complex structure composed of infected RBCs by loading different number of intra-cellular parasite, a condition which lends the parasite population both stability and the ability to respond promptly to diverse external conditions (Figure 1E).

As an apicomplexan parasite, *Babesia divergens* exhibits a unique apical complex composed of specialized cytoskeletal and secretory organelles, including micronemes, rhoptries, and dense granules [31]. This invasion apparatus allows rapid and efficient parasite invasion of the RBC to avoid phagocytic engulfment. It has been shown that the invasion process is extremely rapid and of the order of seconds. Despite this, the *B. divergens* merozoites are also extremely hardy and can successfully invade RBCS even minutes after being extracellular, unlike the more fragile malaria merozoites [32]. A recent study showed that the invasion time recorded for each free merozoite was different and was mainly influenced by the time for the pre-invasion phase [33]. The invasion process was completed on average in 72.2 s and the shortest and longest time taken for merozoites to invade the erythrocyte was 38 s and 126 s, respectively. The pre-invasion phase time was estimated from the release of the free merozoite to the establishment of the tight connection with the erythrocyte plasma membrane and took on average 41.7 s [33].

Invasion of red blood cells (RBCs) is one of the critical points in the lifecycle of Babesia [31]. The parasite does not invade other host cells (Figure 1D). This specificity implies the presence of receptor(s) on the erythrocyte that is recognized by a complementary molecule(s) on the parasite. Experimental manipulation of human RBCs in vitro has shown that *B. divergens* has multiple routes of entry into the RBC, as enzymatic treatment of RBCs with diverse proteolytic enzymes does not abolish parasite invasion [29]. This is a remarkable parasite feature given the relatively recent association of Babesia with the human host RBC. On the other hand, because of Plasmodium’s long and involved history with man, RBC proteins associated with Plasmodium invasion constitute primary targets of malaria parasite-induced selection, and, in fact, almost all examples of molecular evolution in humans (e.g., sickle-cell anemia, Duffy-negativity, G6PD deficiency) are attributable to selection for polymorphisms that reduce susceptibility to severe malaria [34]. Thus, malarial invasion ligands have evolved to keep pace with the changes in human host receptor repertoire. As babesiosis is a zoonosis and has had to adapt to RBCs from multiple species exhibiting RBC of very different molecular architectures, this ability to successfully invade RBCS that have been denuded of different receptors may not come as a total surprise. It has been experimentally shown that *B. divergens* does not rely on a single receptor for host cell entry [29]. This ensures that the parasite when faced with RBC antigenic polymorphism would still be able to mount an efficient attack on the RBC and successfully propagate. Thus, a molecular repertoire of ligands that can bind to multiple surface receptors of diverse RBCs allows the parasite to persist even in heterogenous human populations.

## 5. *Babesia divergens* Flexible Intra-Erythrocytic Life Cycle Leads to Pleomorphic Life Forms

The development of synchronization methods for *B. divergens* allowed the detailed analysis of life cycle parameters of the parasite [30]. Surprisingly, it has been found that unlike other Apicomplexan parasites, population control measures are adopted by the parasite in which, at high parasitemia, options to prolong the RBC cycle are taken, whereas at lower parasitemia, exiting the RBC is chosen to invade new cells and increase population numbers. This developmental choice is unique for *Babesia* compared with other Apicomplexans like *P. falciparum* and *Toxoplasma gondii*, which have precisely timed intracellular cycles. *P*. *falciparum* typically spends 48  h in each erythrocytic cycle, while tachyzoite of *T*. *gondii* has a 6 h growth and replication period inside the host cell before egress [35]. Multiple definitions of the intra-erythrocytic lifecycle for *B. divergens* have been laid out in keeping with the complex morphological presentation of the parasites inside the RBC. Historically, an 8 h lifecycle was attributed to the parasite, although no clear rationale for this important chronological hallmark had been laid out [36]. A 4h life cycle [37] has also been proposed for *B. divergens*, within the RBC, based on the first events of egress. Data from other laboratories confirm that 4–5 h is an accurate representation of the shortest cycle of the parasite [30]. Studies from our group have outlined the developmental options available to the asexual Babesia parasite in the RBC, reporting a sequential progression of the seven morphological or pleiomorphic forms of *B. divergens* in culture (Figure 1E) and showed that the dynamics of parasite proliferation and differentiation that are maintained through controls that secure the different infected RBC populations in strict proportions. Using a more complex definition of the cycle of the parasite population as a whole, the asexual cycle can be defined as the time required for a manifestation of all 7 morphogenetic forms of the parasite together with clear periods of parasite egress followed by new host cell invasion. Using this parameter, 24 h is the time needed for the parasite population to complete these events [30]. Thus, the flexibility of this phase of the *B. divergens* lifecycle allows for parasite persistence, depending on specific developmental choices that it adopts.

## 6. Maintenance of *Babesia divergens* Population Structure to Enable Quick Adaptation to Changing Environment through Egress and the Release of Different Numbers of Infective Units

**Figure 2:**
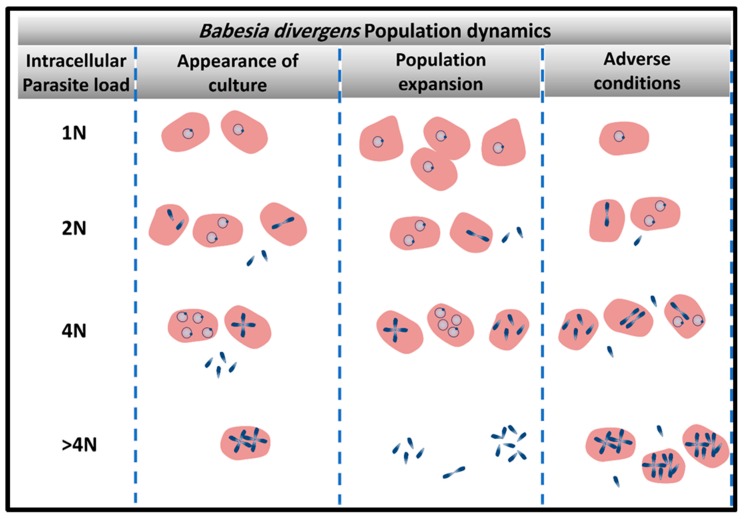
Persistence of parasite infection is dependent on the dynamics of parasite population. The heterogenous structure of the parasite population is a strategy exploited by the parasite for survival. Four main populations of infected red blood cells (RBCs( (1N-, 2N; 4N-and > 4N-iRBCs) vary in frequency based on the population needs. Under population expansion, >4N-RBC are less frequent since there is no need for the storage of infective merozoites. Under adverse conditions, multiple parasites are arrested within the host cells until the environmental condition is restored.

The survival of the parasites in a hostile environment is achieved by the regulation of the structure of its population. Persistence is dependent on the structure and the dynamics of movement between parasite sub-populations (Figure 2). Merozoites are the only parasite form that can egress to allow new invasion resulting in rise in parasitemia. However, these infective units can arise from multiple parasite reservoirs like the paired figure or the Maltese Cross, or two paired figures or the multiple parasites populations [30]. Depending on the favorable conditions for population expansion, these infective units are released to initiate new infective cycles (Figure 1F). If there is an urgent need for culture expansion, two merozoites will be released from the RBC, diverting the parasite away from differentiation of double trophozoites into double paired figures or paired figures into Maltese Cross formation. Despite the apparent heterogeneity of the parasite populations that encompasses seven forms, the basic population is composed of mainly 4 sub-populations of RBC (based on the number of genomes “N”: 1N-; 2N-; 4N-and >4N- iRBCs; defined as “intracellular parasite load”) which individually follow synchronized paths, tightly controlled by the ratios of invasion, development, and egress based on prevailing environmental conditions [30] (Figure 2). Parasite populations can assume the pattern of “exponential expansion” under optimum conditions based on the availability of adequate nutrients and RBCs that can serve as hosts. In this case, 1N-, 2N, and 4N-iRBCs fluctuate in frequencies based on sequential cycles of egress and invasion [30]. Under this dynamic, >4N-RBC are less frequent since there is no need for storage of infecting parasites (Figure 2). However, under adverse conditions the parasite population can interrupt the normal processes of invasion and development/proliferation by maintaining the 1N- and 2N-infected RBC sub-populations at similar frequency while inhibiting egress and thus, preserving a higher frequency of 4N- and >4N-iRBCs is seen.

The parasite also exhibits additional adaptive mechanisms when confronted with stress, involving changes in the population structure, with differential adjustments among the ratios of the infected sub-RBC populations as a function of the strength of the stress [32]. When exposed to drugs and toxic chemicals like the calpain inhibitor and allantoin, RBCs harboring multiple parasites are the most affected parasite populations and their numbers visibly decline in the population. Parasites developed into crisis forms, and RBCs infected by multiple parasites became less frequent, almost disappearing at the end of the long treatment. In contrast, the percentage of 1N-RBCs progressively increased in culture with an increase of stress severity. Thus, *B. divergens* is able to alter its population structure favoring those sub-populations like the 1N population, which is able to withstand the stress and return the population to its normal heterogenous state, once the stress has been removed [32]. This strategy once again allows parasite persistence even when drug pressure is applied.

## 7. Persistence and Implications for Pathogen Inactivation Strategies

The strategy of storing protected merozoites within the erythrocyte guarantees a ready arsenal for new invasion processes when optimal conditions are restored [32]. If the optimal conditions are not restored, as mentioned before, the parasitemia progressively decreases over time. *B. divergens* has a high proliferative potential; therefore, if not completely exterminated, the few remaining parasites can restart a new population, representing a huge threat for the development of efficient pathogen reduction methods, because reduction in the population does not guarantee absence of infection in immuno-compromised patients. This has particularly crucial implications for blood banks as storing infected RBCs for a prolonged period demonstrates that the parasites can persist despite steep losses in initial numbers [38]. After 24 h of storage at 4 °C, a substantial reduction of parasites was shown in the blood bags, which was maintained throughout storage. This decrease was accompanied by a change in morphology of parasites, with the number of altered parasites increasing through the period of storage. However, parasite viability was maintained through 31 days of cold storage with a lag in achieving exponential growth seen in the parasites subjected to longer periods of refrigeration [38]. Importantly, these results show that even a small number of infectious parasites that persist through adverse conditions can lead to large populations of parasites in a relatively short period of time.

## 8. Conclusions

The life cycle of *Babesia divergens* includes specific strategies required for the adaptation to various types of host cells and to the different environments that may confront the parasite-both supportive and hostile. A better understanding of all aspects of the battle of persistence between *Babesia* and its hosts will be useful for the development of better strategies and drugs to control the parasite.
